# Supplementary material for: Sinus venosus adaptation models prolonged cardiovascular disease and reveals insights into evolutionary transitions of the vertebrate heart
Source: Nat Commun. 2023 Sep 7;14:5509. doi: 10.1038/s41467-023-41184-y (PMC10485058; doi:10.1038/s41467-023-41184-y)
Supplement: Supplementary file 1 — Supplementary Information [file 41467_2023_41184_MOESM1_ESM.pdf]

Supplementary information

Gafranek et al. Sinus venosus adaptation models prolonged cardiovascular disease and reveals insights into vertebrate heart evolutionary transitions

Contents:

Supplementary Figs. 1-22

Supplementary Tables 1 and 2.

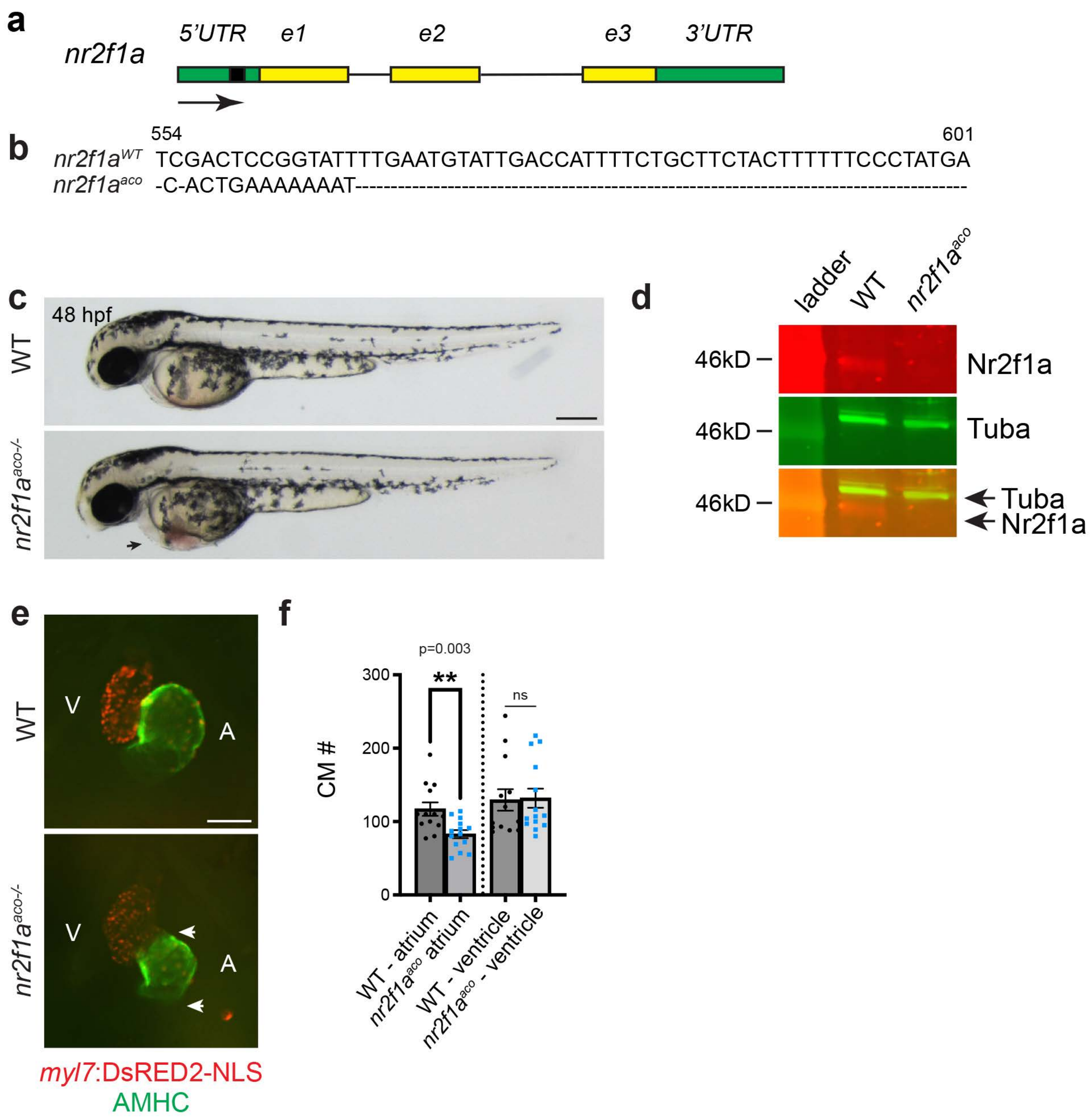

Supplementary Fig. 1. The *aco* mutation is an *nr2f1a* mutant allele. a. Schematic of *nr2f1a* transcript showing 5'- and 3'-UTRs (green), translated portion of exons (yellow), and introns (black lines). Black box indicates location of deletion. **b.** WT and *nr2f1a^{aco}* allele sequence showing the 45 bp deletion (58 bp deletion and 13 bp insertion) within the 5'-UTR. **c.** Lateral view of *nr2f1a^{aco}* mutants at 48 hpf. Arrow indicates the moderate pericardial edema and blood pooling. Anterior is left. Dorsal is up. Scale bars - 200 µm **d.** Western blot showing the loss of Nr2f1a in *nr2f1a^{aco}* mutants. Source data are provided as a Source Data file. **e.** Frontal views of hearts from WT sibling and *nr2f1a^{aco}* mutants showing the morphologically smaller atria of the mutants. The arterial pole is upward. V indicates ventricle. A indicates atrium. Scale bar - 50 µm. **f.** Quantification of atrial and ventricular CMs shows there is a specific reduction in atrial CMs at 48 hpf. WT n=13, *nr2f1a^{aco}* n=14. All embryonic phenotypes for the *nr2f1a^{aco}* were equivalent to previously reported *nr2f1a* mutant alleles[38]. An unpaired, two-sided Student's t-test was used to assess statistical differences between WT and *nr2f1a^{aco}* atrial and ventricular cardiomyocytes. Comparison marked ** denotes p=0.003. Error bars represent the mean +/- SEM. Source data are provided as a Source Data file.

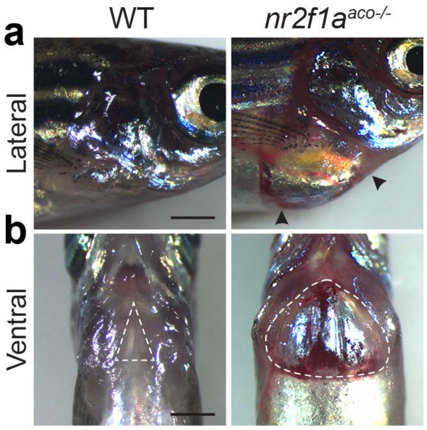

Supplementary Fig. 2. Overt cardiac distress in adult *nr2f1a^{aco}* mutants. a. Brightfield images of anterior-lateral profiles of adult WT and *nr2f1a^{aco}* mutant fish. Anterior is right. Dorsal is up. Black arrowheads indicate the extent of the pericardial edema in the mutant. **b.** Brightfield images showing ventral views of the same fish in a. Dashed lines denote the perimeter of the thoracic cavity containing the hearts. Regardless of the development and extent of pericardial edema, all *nr2f1a^{aco}* mutant hearts are enlarged and have similar morphology. Scale bars - 1 mm.

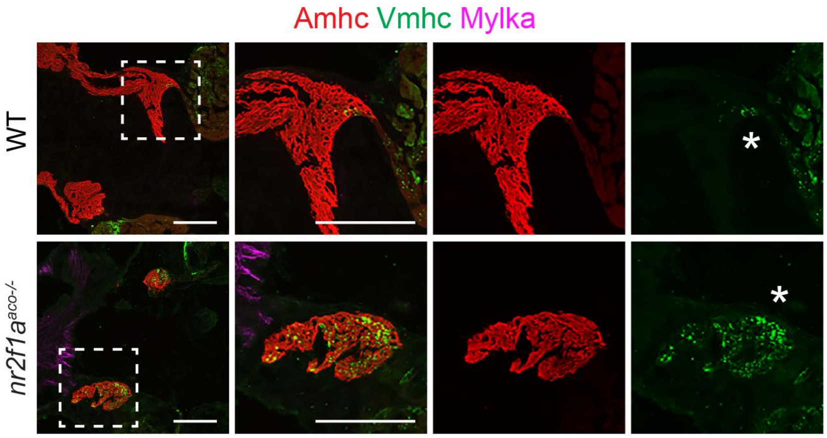

Supplementary Fig. 3. ***Nr2f1a*** **mutant hearts lack atrial tissue and co-express ventricular markers.** Confocal images on sections of hearts from the border of the atrial and ventricular chambers in adult WT and *nr2f1a*^{aco} mutant hearts immunostained for Amhc (red), Vmhc (green), and Mylka (magenta). All the Amhc+ CMs in *nr2f1a*^{aco} mutant hearts co-express Vmhc, while this co-expression in WT hearts is restricted to a small set of cells within the atrioventricular canal. Insets indicate higher magnification images presented in the different channels. White asterisks indicate where co-expression of Amhc and Vmhc was found. Scale bars - 100 μm.

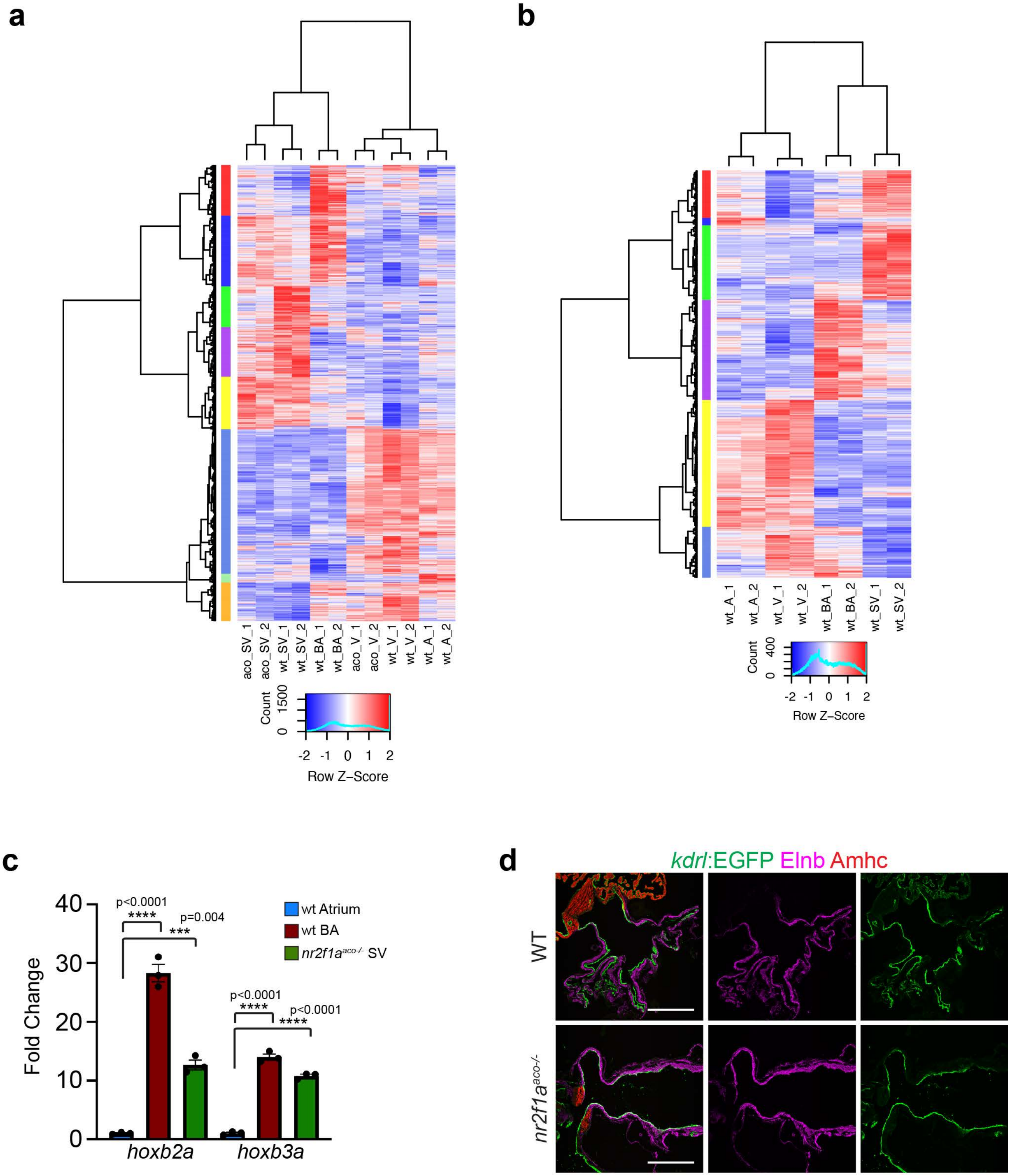

Supplementary Fig. 4. Clustering analysis and validation of markers in the BA and SV of WT and *nr2f1a* mutant fish. a. Hierarchical clustering analysis of bulk RNA-seq from the BA, SV, V, and A of adult WT and *nr2f1a^{aco}* mutant hearts. **b.** Hierarchical clustering analysis of the bulk RNA-seq from the BA, SV, V, and A of the adult WT hearts. **c.** RT-qPCR quantifying the expression of *hoxb2a* and *hoxb3a* in WT BA, *nr2f1a^{aco}* mutant SV, and the WT atrium (control). These hox genes are expressed in the BA and SV, but absent from the atrium. Statistical significance between tissues was calculated using an ordinary one-way ANOVA with multiple comparisons. Comparison marked *** denotes p=0.004. Comparisons marked **** denote p<0.0001. Error bars represent the mean +/- SEM. Source data are provided as a Source Data file. **d.** Confocal images of sagittal sections immunostained for *kdrl:*EGFP (green), Elnb (magenta), and Amhc (red). ECs line the SV of WT and *nr2f1a^{aco}* mutants. This lining is not disrupted in *nr2f1a^{aco}* mutants. WT n=3, *nr2f1a^{aco}* n=2. Scale bars - 200 µm

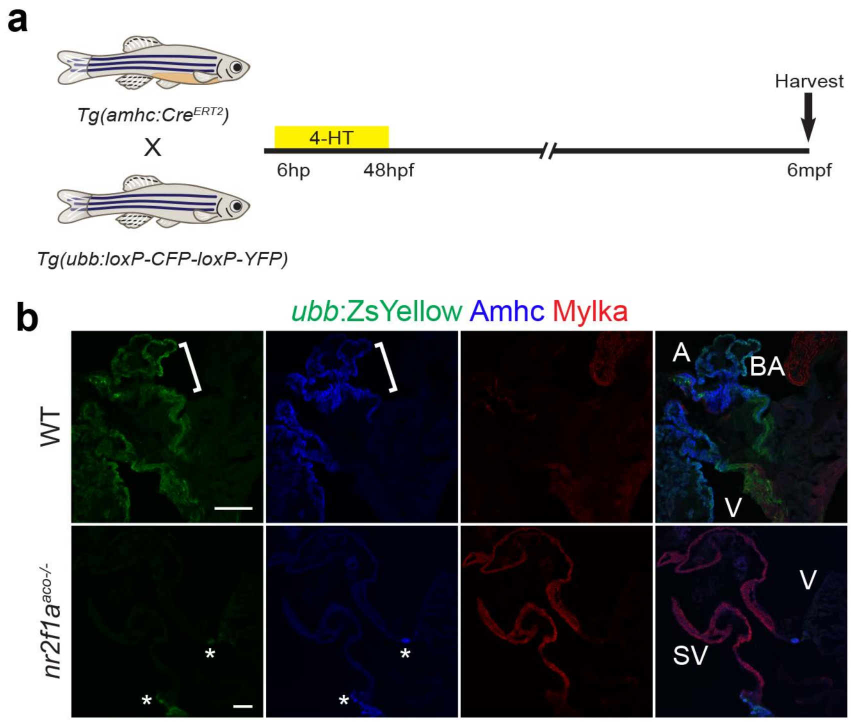

Supplementary Fig. 5. Embryonic atrial CMs do not contribute to the WT or *nr2f1a*^{aco} mutant SV. a. Schematic of the lineage tracing with 4-HT treatment of WT and *nr2f1a*^{aco} mutant embryos with the *Tg(amhc:CreERT2)*; *Tg(ubb:CSY)* transgenes. **b.** Confocal images of sagittal sections from adult WT and *nr2f1a*^{aco} mutant hearts immunostained for atrial ZsYellow (green), Amhc (blue), and Mylka (red). White brackets mark the area of overlapping lineage-traced atrial CMs in the adult atria. White asterisks indicate remnant Amhc+ CMs in the adult hearts that were derived from the embryonic atrial CMs. Distal cells in the SV were not labeled in WT or *nr2f1a*^{aco} mutant hearts, suggesting embryonic atrial CMs do not invade or contribute to the WT SV and that transdifferentiation of embryonic atrial CMs does not contribute to the SV defects. WT and *nr2f1a*^{aco} n=3 Scale bars - 100 μm

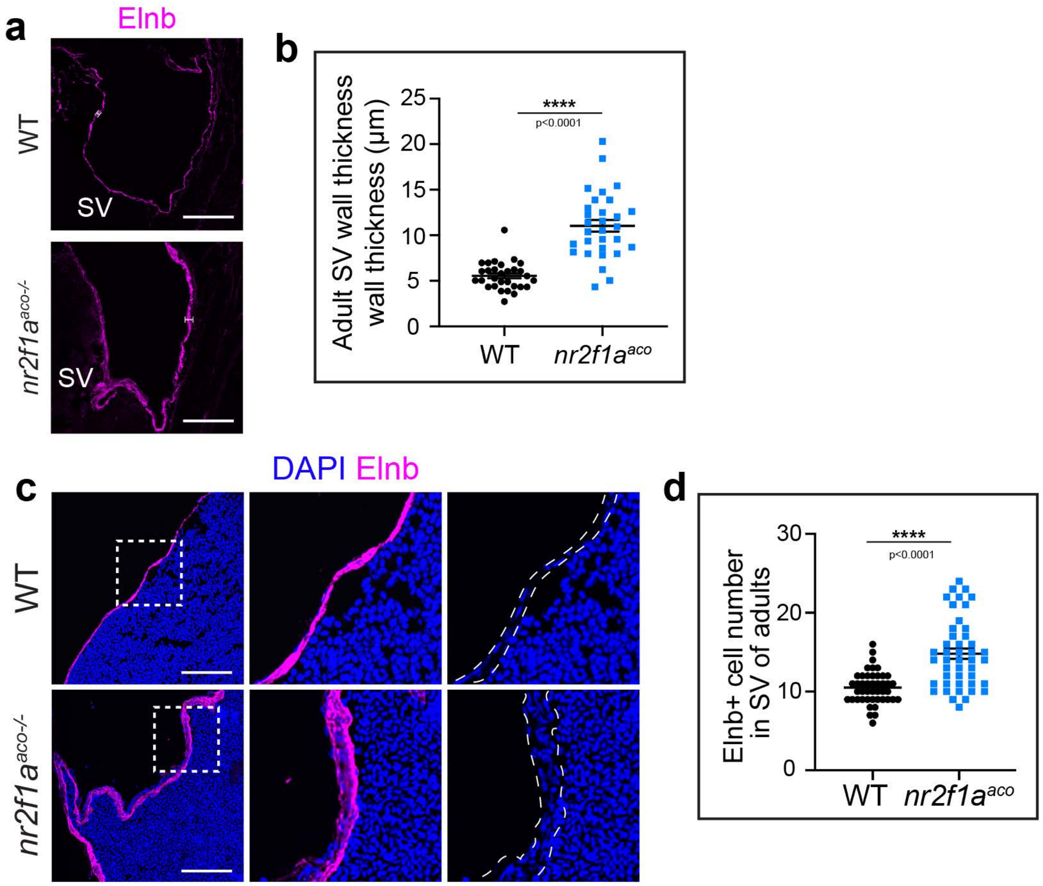

Supplementary Fig. 6. Adaptive remodeling in the adult SV. a. Confocal images of sagittal sections from adult WT and *nr2f1a^{aco}* mutant SV immunostained for Elnb (magenta). Bars indicate thickness of the SV. Scale bars - 100 µm. **b.** Quantification of Elnb thickness in the sections of the adult WT and *nr2f1a^{aco}* mutant SV (n=10 fish per group). Individual points on the graph represent 10 measurements averaged from different regions of the SV in a single section (3 total sections per fish). **c.** Confocal images of sagittal sections of the adult WT and *nr2f1a^{aco}* mutant SV immunostained for Elnb (magenta) and DAPI (blue). Scale bars - 100 µm. **d.** Quantification of cells (indicated by DAPI+ nuclei) within the Elnb of the adult WT and *nr2f1a^{aco}* mutant SV (n=3 fish per group). Individual points on the graph represents 1 randomly chosen region within the SV for counting (5 regions per section were counted from 3 sections per fish). The data support there are more cells within the thicker Elnb+ layer of the SV. An unpaired, two-sided Student's t-test was used to assess statistical differences between WT and *nr2f1a^{aco}* (b and d). Comparisons marked **** denote $p<0.0001$. All error bars represent the mean +/- SEM. Source data are provided as a Source Data file.

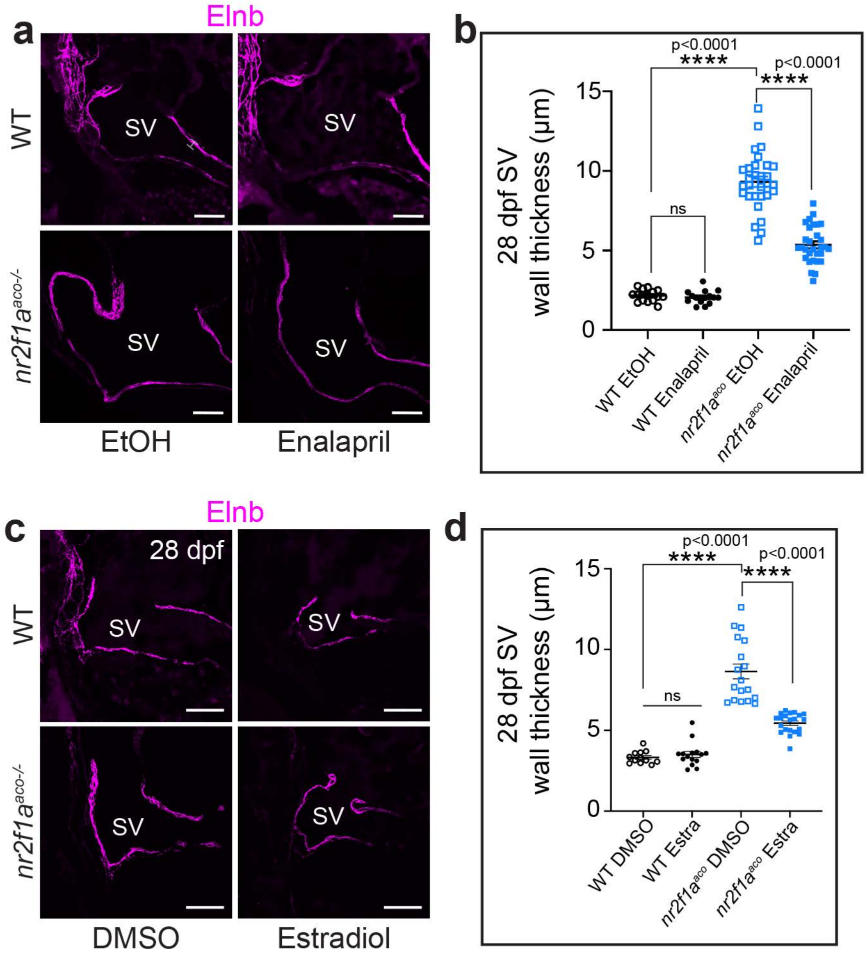

Supplementary Fig. 7. Prolonged treatment with vasodilators reduces the thickening of the SV observed in *nr2f1a* mutants. a. Confocal images of sagittal sections from 28 dpf WT and *nr2f1a*^{aco} mutant fish treated with either Enalapril or ethanol (control). Scale bars – 50 µm. **b.** Quantification of Elnb+ wall thickness at 28 dpf following Enalapril treatments. Individual points on the graph represent 10 measurements averaged from different regions of the SV in a single section (3 total sections per fish). WT control and treated n=5, *nr2f1a*^{aco} control and treated n=10. **c.** Confocal images of sagittal sections from 28 dpf WT and *nr2f1a*^{aco} mutant fish treated with either estradiol or DMSO (control). Scale bars – 50 µm. **d.** Quantification of Elnb+ wall thickness at 28 dpf following estradiol treatments. Individual points on the graphs represent 10 measurements averaged from different regions of the SV in a single section (3 total sections per fish). WT control n=4, WT treated n=5, *nr2f1a*^{aco} control n=6, *nr2f1a*^{aco} treated n=7. Statistical significance between treatment groups was calculated using an ordinary one-way ANOVA with multiple comparisons in b and d. Comparisons marked **** denote p<0.0001. All error bars represent the mean +/- SEM. Source data are provided as a Source Data file.

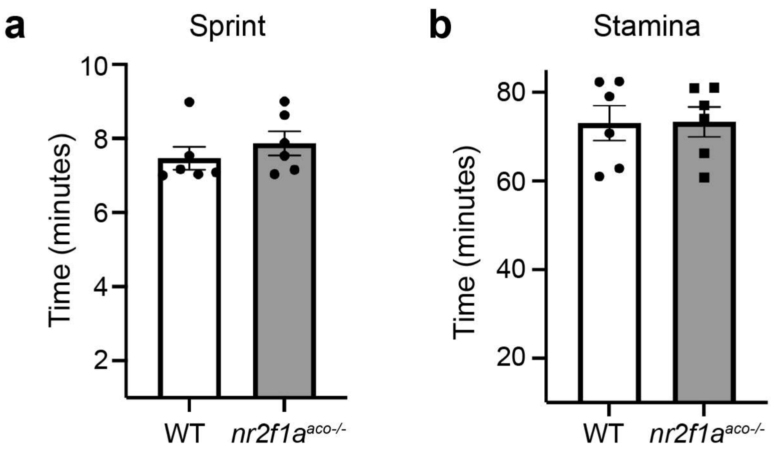

Supplementary Fig. 8. Performance of adult WT and *nr2f1a^{aco}* mutant fish is equivalent in swim tests. a. Time it took for adult WT and *nr2f1a^{aco}* mutant fish to fail in the sprint swimming test. **b.** Time it took for adult WT and *nr2f1a^{aco}* mutant to fail in the endurance swimming test. Differences were not significant. Thus, despite the adaptive remodeling of the SV and enlargement of the heart, the basic performance of the adult fish is not affected in the *nr2f1a^{aco}* mutant fish. n=6 fish examined per test. An unpaired, two-sided Student's t-test was used to assess statistical differences in exercise performance between WT and *nr2f1a^{aco}*. All error bars represent the mean +/- SEM. Source data are provided as a Source Data file.

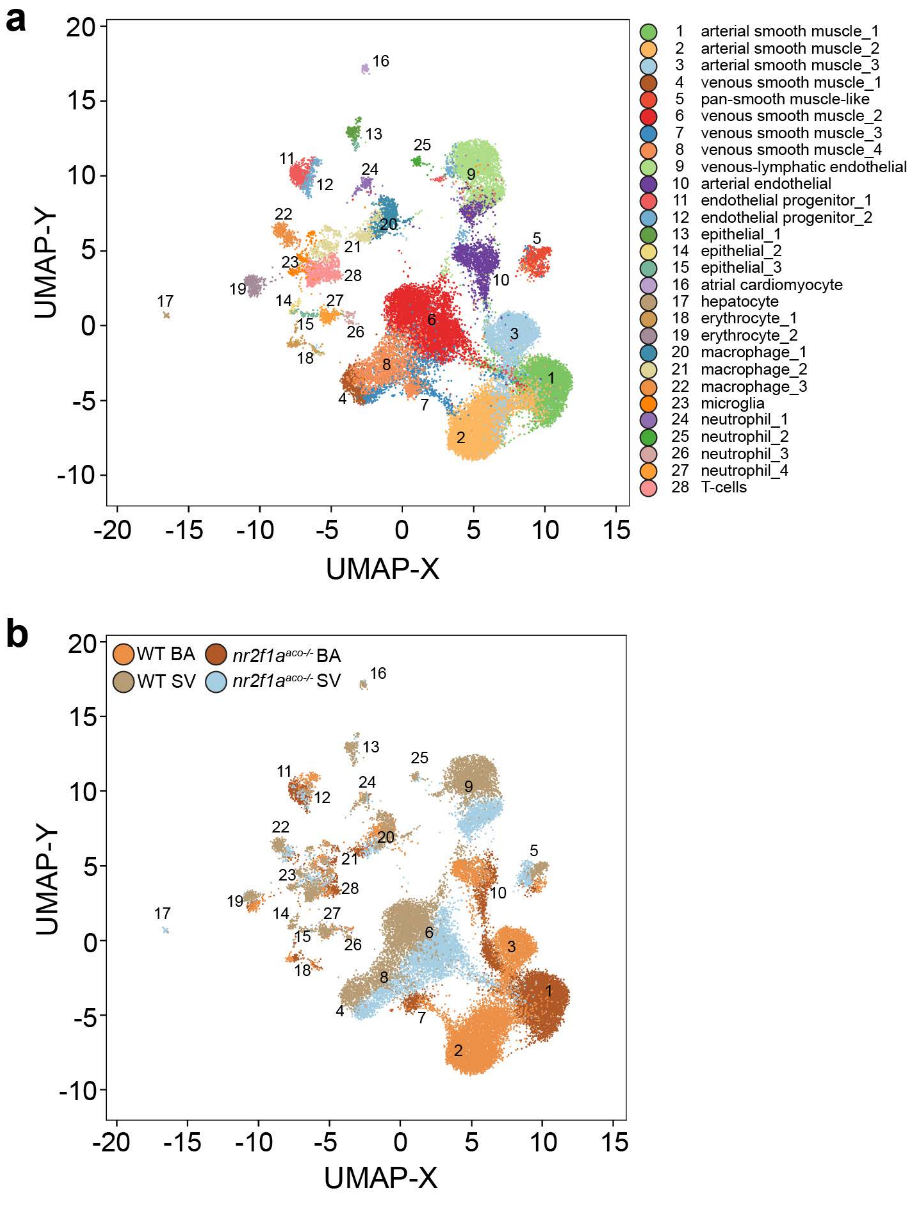

Supplementary Fig. 9. Total scRNA-seq clusters of the BA and SV in WT and $nr2f1a^{aco}$ mutant fish. a. UMAP of all the clusters (1-28) from the scRNA-seq of the BA and SV in WT and $nr2f1a^{aco}$ mutant fish. Differences in blood cells and other small contaminate populations were not found. **b.** UMAP data showing the contributions to all 28 clusters from the BA and SV of WT and $nr2f1a^{aco}$ mutant fish.

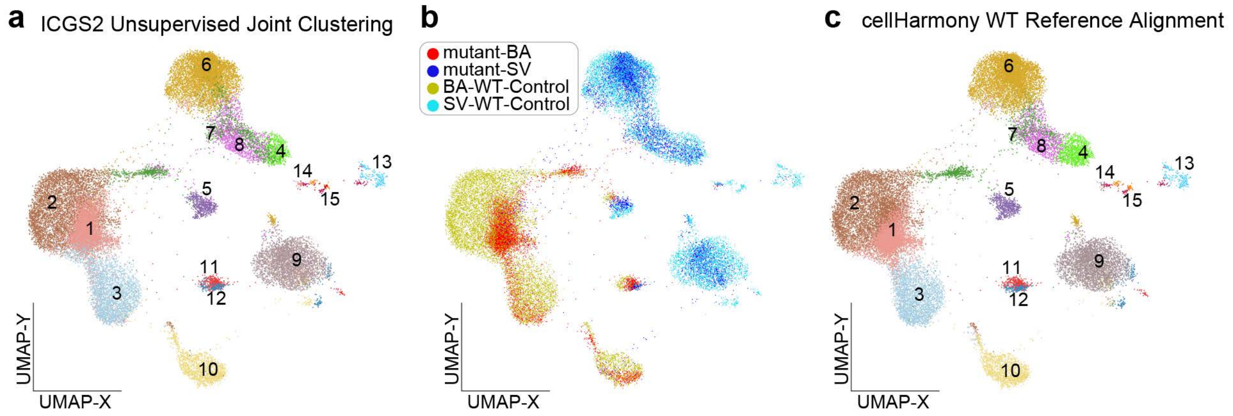

Supplementary Fig. 10. Supervised assignment of clusters based only on WT cells. a. UMAP of clusters 1-15 with the *nr2f1a*^{aco} mutant cells and ICGS2 cluster identities projected onto the WT-only cell embedding (UMAP-transform, all WT and mutant cells displayed). **b.** UMAP of clusters 1-15 with the *nr2f1a*^{aco} mutant clusters projected upon the WT clusters showing the origins of the cells from the unsupervised projection. **c.** UMAP of clusters 1-15 with the *nr2f1a*^{aco} mutant cells aligned (cellHarmony) with the WT samples as a reference, as an independent indicator of cell-type identity.

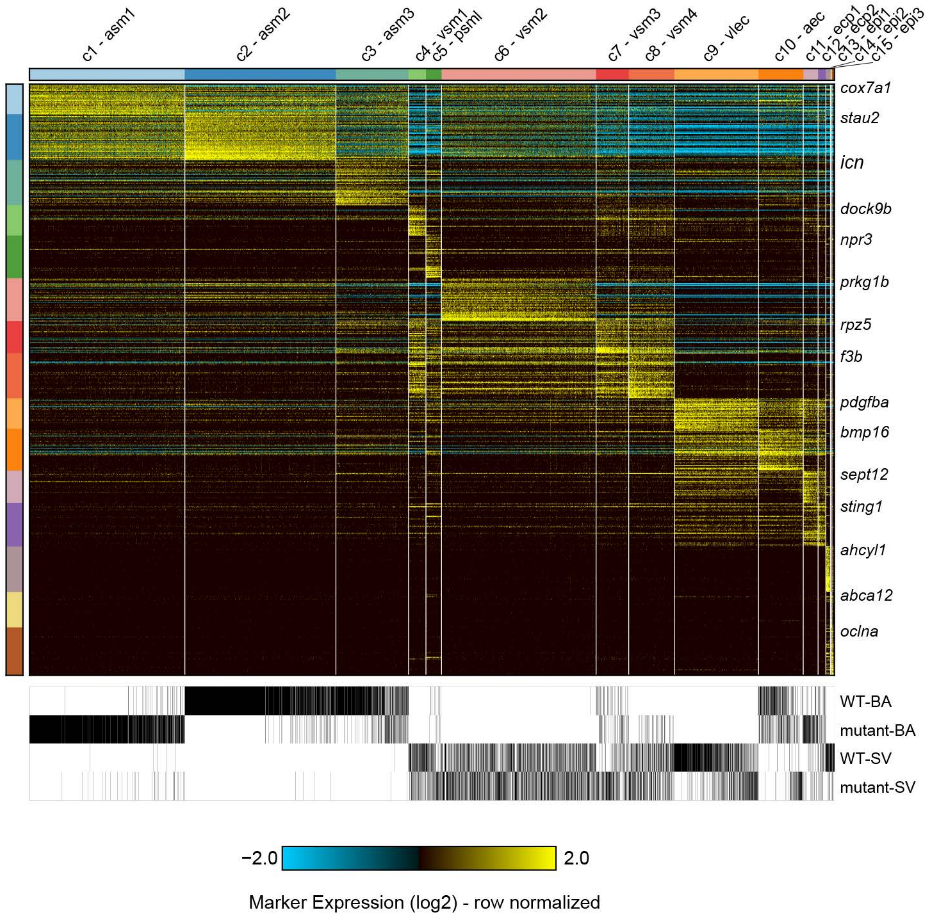

Supplementary Fig. 11. Marker gene expression of scRNA-seq clusters from the BA and SV of adult WT and *nr2f1a^{aco}* mutant fish. Heatmap of the top marker genes (MarkerFinder algorithm) for the 15 filtered ICGS2 cell populations from the combined scRNA-seq analysis.

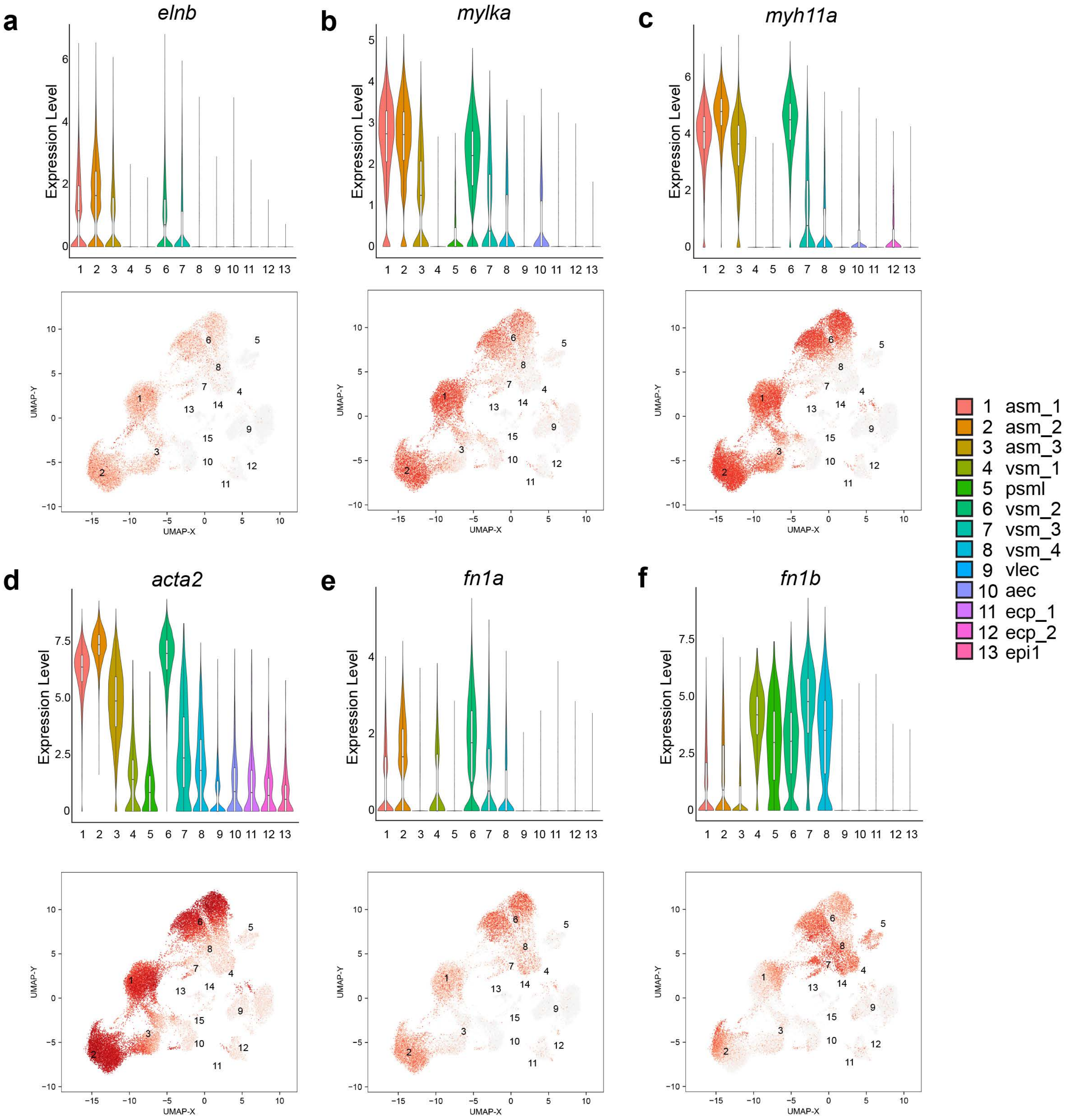

Supplementary Fig. 12. Expression of SMC markers in the BA and SV. a-f. Expression of *elnb*, *mylka*, *myh11a*, *acta2*, *fn1a*, and *fn1b* in violin plots for clusters (1-13) (n = number of cells per cluster (sum of WT and $nr2f1a^{aco}$ mutant cells per cluster) indicated in Supplementary Data 5 for clusters 1-13) and in UMAPs for clusters 1-15. *Elnb*, *mylka*, *myh11a*, and *acta2* are expressed at high levels in clusters 1-3, and 6 and lower levels in clusters 4, 7, and 8. Expression of *fn1a* and *fn1b* are expressed at relatively higher levels in clusters 4, 7, and 8, as well as 1, 2, and 6. The box plot indicates the median; and the lower and upper hinges indicate the 25th (Q1) and 75th (Q3) percentile, respectively. The whiskers indicate the standard outlier definitions (upper whisker = Q3 + 1.5 (Q3-Q1) and lower whisker = Q1 – 1.5 (Q3 – Q1)).

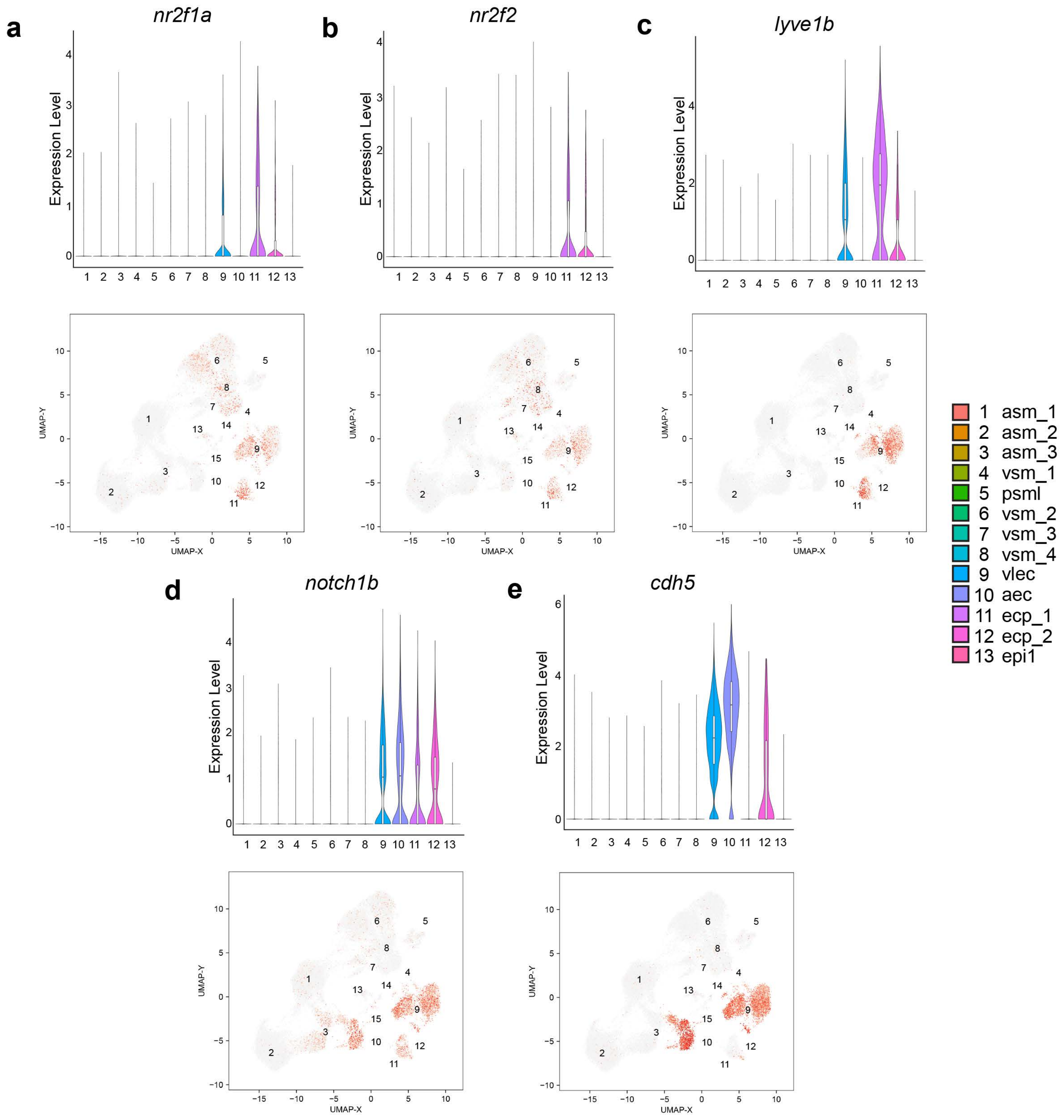

Supplementary Fig. 13. Expression of arterial and venous EC marker genes in the BA and SV. a-e. Expression of *nr2f1a*, *nr2f2*, *lyve1b*, *notch1b*, and *chd5* in violin plots for clusters (1-13) (n = number of cells per cluster (sum of WT and *nr2f1a*^{aco} mutant cells per cluster) indicated in Supplementary Data 5 for clusters 1-13) and in the UMAPs for clusters 1-15. *Nr2f1a*, *nr2f2*, and *lyve1b* are expressed primarily in venous-lymphatic ECs and EC progenitor cells (clusters 9, 11, and 12). *Nr2f1a* and *nr2f2* were also expressed at low levels in smooth muscle cells (clusters 4, 6, 7, and 8). *Notch1b* was expressed throughout arterial and venous ECs and progenitors (clusters 9-12). *Chd5* was expressed throughout arterial and venous endothelial cells (clusters 9 and 10), but excluded from endothelial progenitors (clusters 11 and 12). The box plot indicates the median; and the lower and upper hinges indicate the 25th (Q1) and 75th (Q3) percentile, respectively. The whiskers indicate the standard outlier definitions (upper whisker = Q3 + 1.5 (Q3-Q1) and lower whisker = Q1 – 1.5 (Q3 – Q1)).

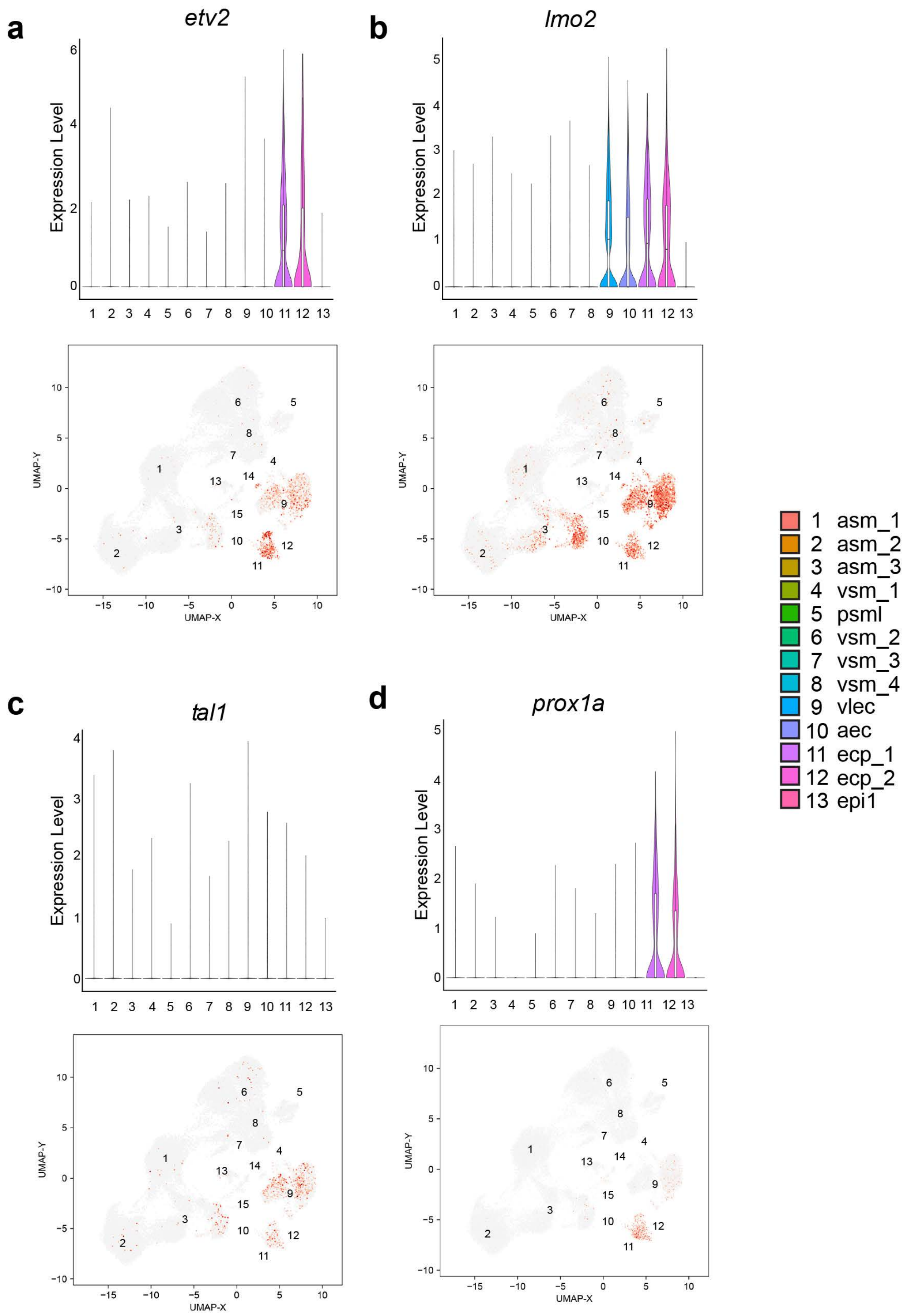

Supplementary Fig. 14. Expression of EC progenitor marker genes in the BA and SV. a-d. Expression of *etv2*, *lmo2*, *tal1*, and *prox1a* in violin plots for clusters (1-13) (n = number of cells per cluster (sum of WT and *nr2f1a*^{aco} mutant cells per cluster) indicated in Supplementary Data 5 for clusters 1-13) and in the UMAPs for clusters 1-15. *Etv2* and *prox1a* are expressed at higher levels in EC progenitors (clusters 11 and 12). *Lmo2* and *tal1* are expressed throughout arterial and venous ECs and EC progenitor (clusters 9-12), although *tal1* has significantly lower expression in these cells. The box plot indicates the median; and the lower and upper hinges indicate the 25th (Q1) and 75th (Q3) percentile, respectively. The whiskers indicate the standard outlier definitions (upper whisker = Q3 + 1.5 (Q3-Q1) and lower whisker = Q1 – 1.5 (Q3 – Q1)).

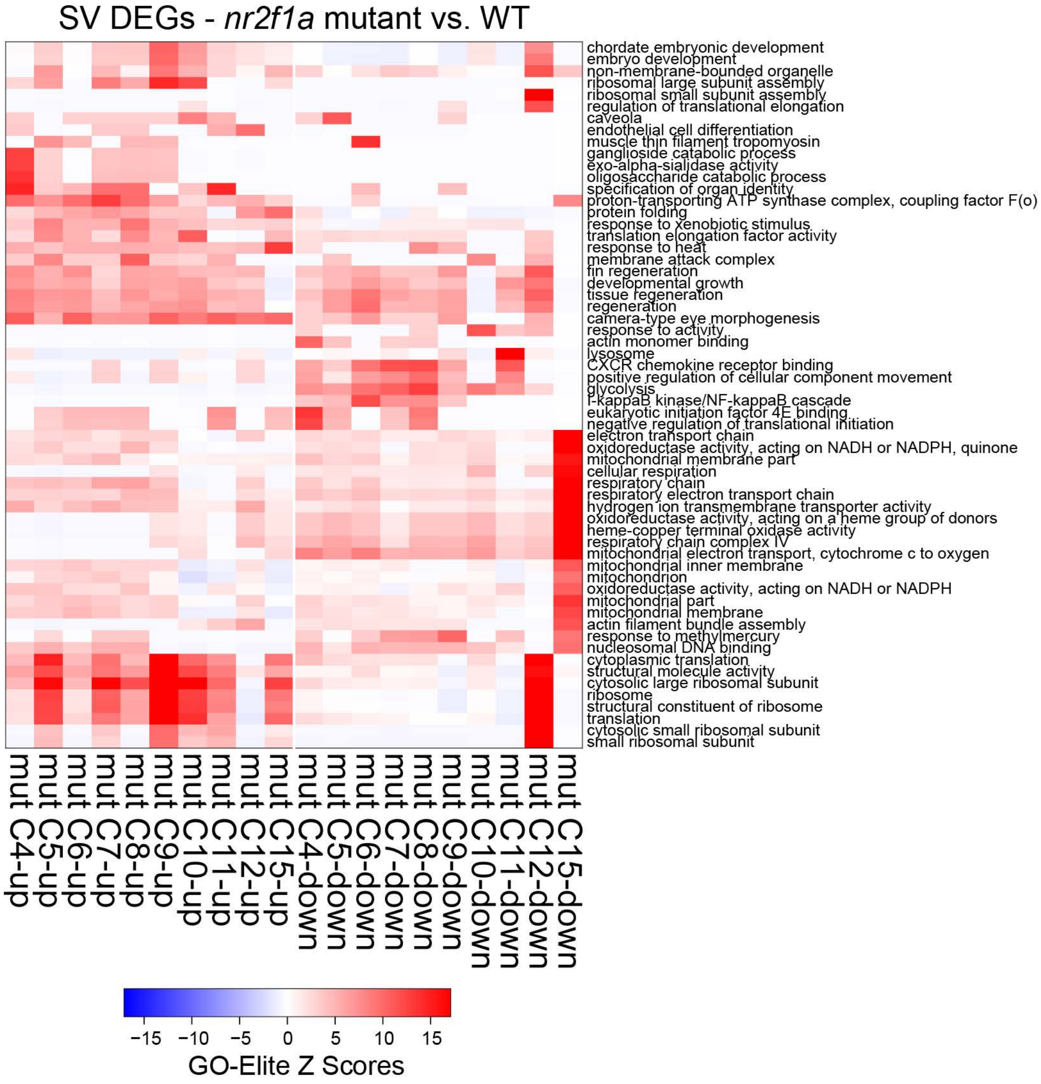

Supplementary Fig. 15. GO enriched terms for *nr2f1a*^{aco} mutant induced gene expression differences in SV. Heatmaps showing GO gene set enrichments (GO-Elite Z-scores) for DEGs (Supplementary Figure 16a) comparing *nr2f1a*^{aco} mutant to WT SV for all 15 ICGS2 cell populations.

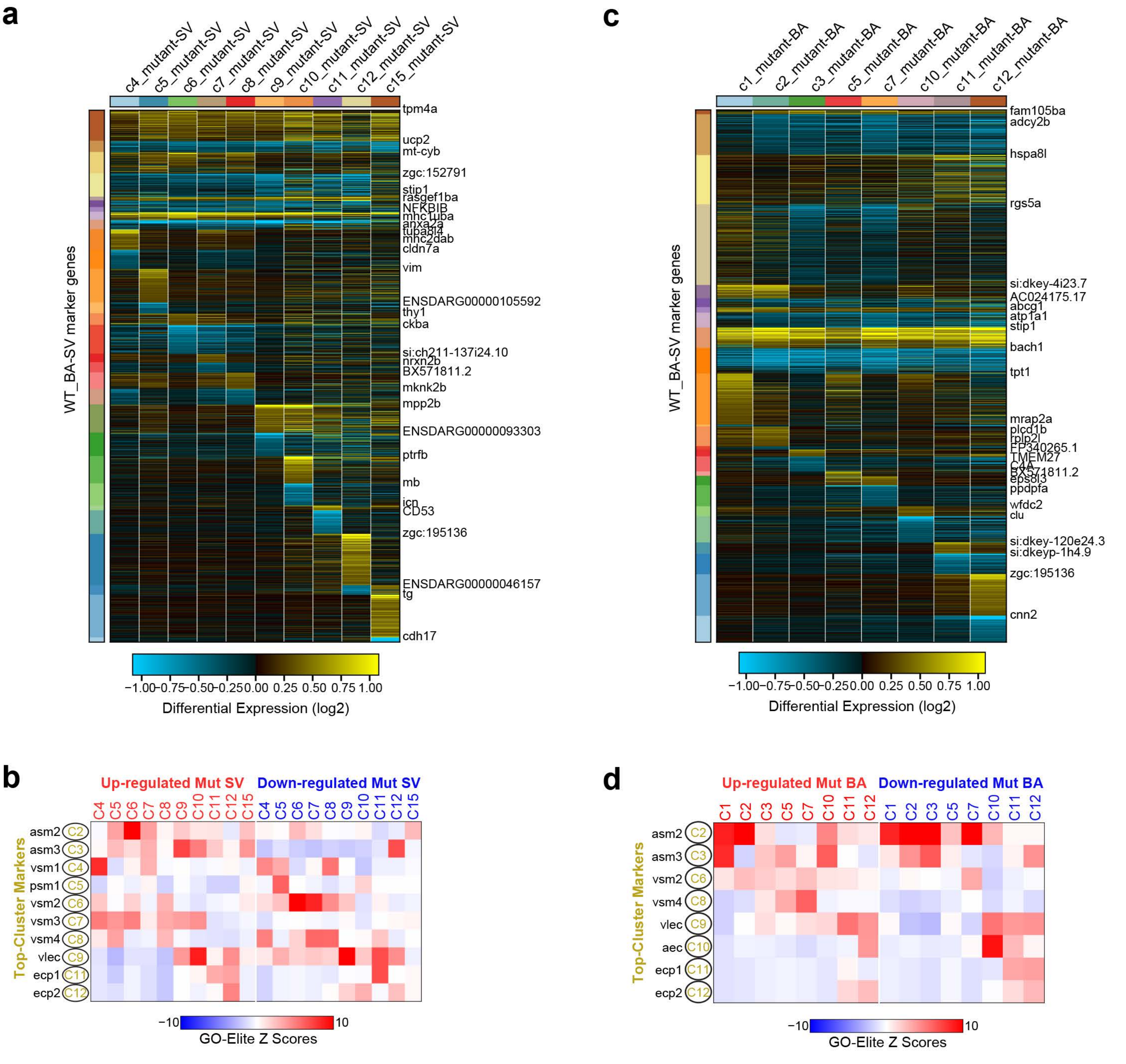

Supplementary Fig. 16. GO enrichment analysis of DEGs from *nr2f1a^{aco}* SV and BA clusters. a, c. cellHarmony heatmaps identified in the *nr2f1a^{aco}* mutant versus WT SV and BA cell populations. DEGs are organized automatically by cellHarmony into predominant patterns of shared and unique gene expression. b, d. GO-Elite heatmaps showing enriched Z-scores for up- or down-regulated genes in the *nr2f1a^{aco}* mutant genes versus WT compared for (b) SV and (d) BA, for the top-200 markers for all WT cell-clusters.

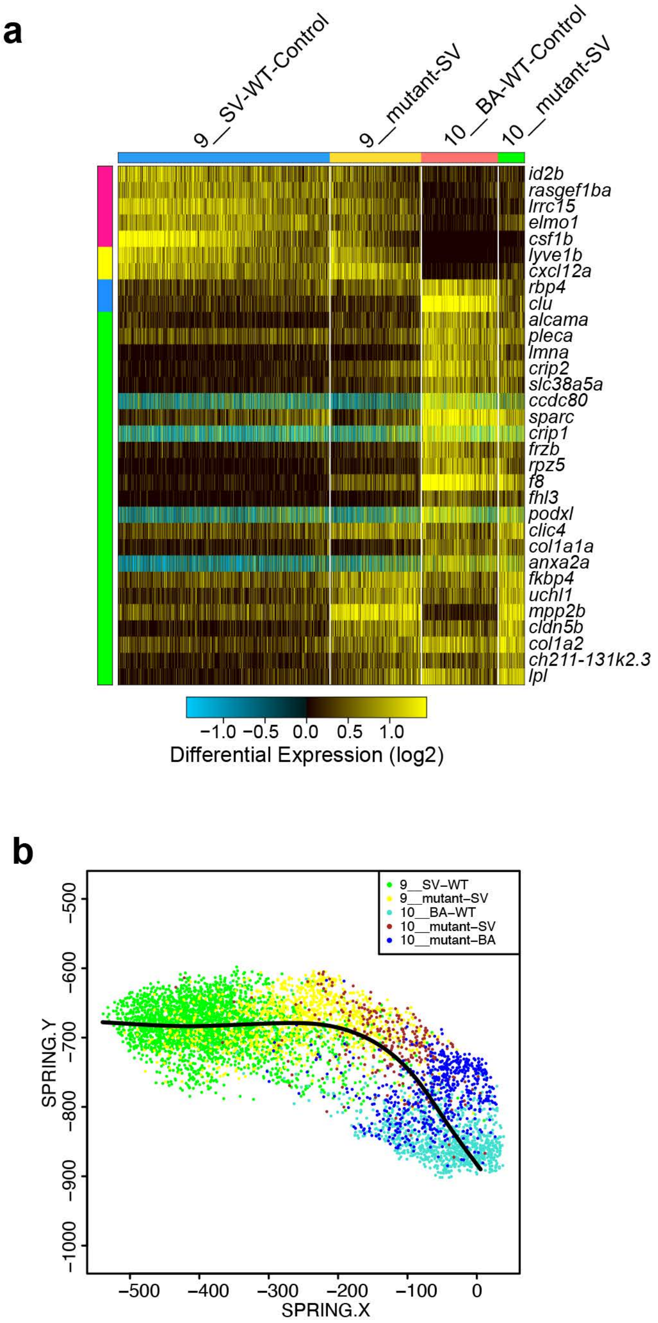

Supplementary Fig. 17. ECs within the *nr2f1a*^{aco} mutant SV have arterial-like gene expression. a. Heatmap showing gene expression from marker genes in the venous (C9) and arterial (C10) EC clusters from the *nr2f1a*^{aco} mutant and WT BA and SV. The C10 ECs from the *nr2f1a*^{aco} mutant SV has similar gene expression to the WT BA C10 ECs. The C9 ECs from the *nr2f1a*^{aco} mutant SV, while still more similar to the WT SV C9 ECs, does have increased expression of some arterial genes. **b**. SPRING plot visualization with SlingShot analysis showing predicted differentiation states of the C9 and C10 clusters shows that C10 cells from the *nr2f1a*^{aco} mutant SV are similar to arterial C10 cells from the BA, while the C9 *nr2f1a*^{aco} mutant cell are more intermediate.

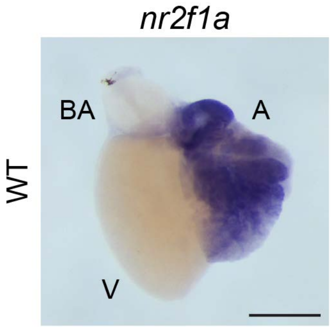

Supplementary Fig. 18. ISH of *nr2f1a* in an adult heart. *Nr2f1a* is expressed in the atrium of a WT adult heart. Atrium (A), Bulbus arteriosus (BA), Ventricle (V). Scale bar - 500 µm.

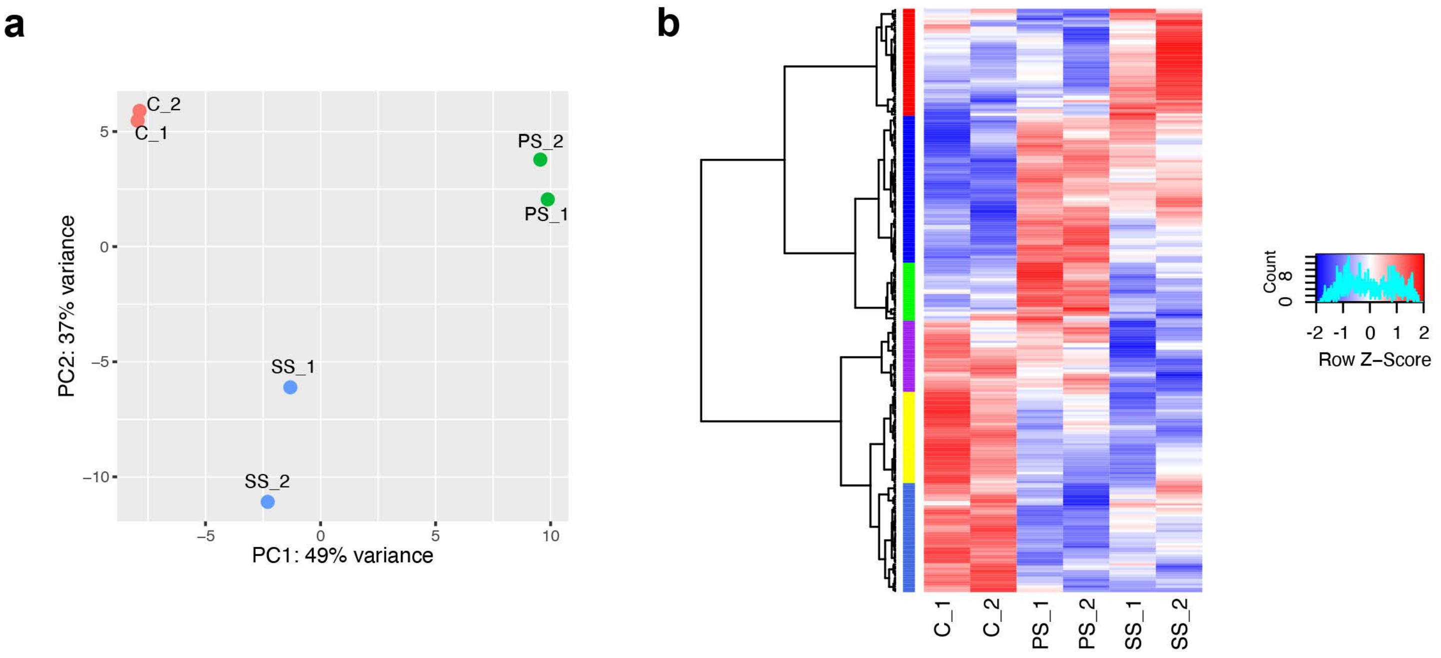

Supplementary Fig. 19. Clustering analysis of *Ciona* heart samples. a. PCA analysis of the different tissues analyzed in the *Ciona* bulk RNA-seq. **b.** Heatmap of hierarchical clustering analysis from the C, PS, and SS samples of the adult *Ciona* hearts.

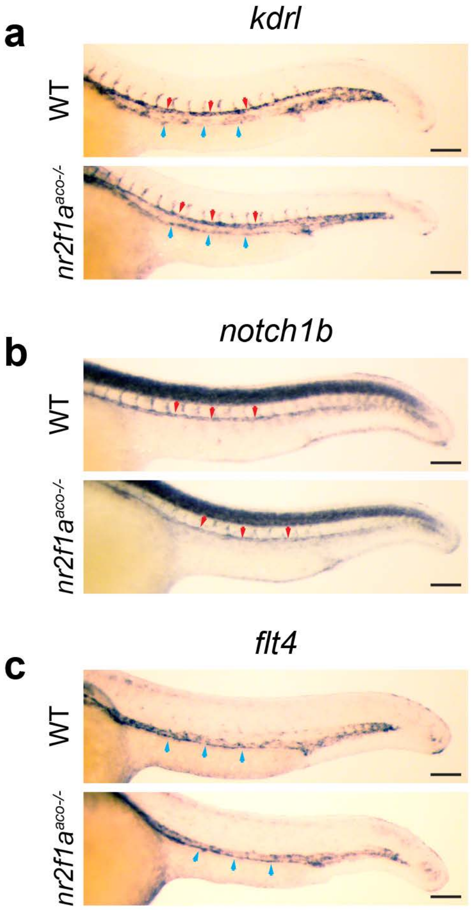

Supplementary Fig. 20. Major vessels are unaltered in *nr2f1a^{aco}* mutant embryos. a-c. The dorsal aorta (red arrowheads) and posterior cardinal vein (blue arrowheads) were not obviously altered in *nr2f1a^{aco}* embryos compared to WT embryos at 24 hpf. *kdrl* (pan-vascular endothelial), *notch1b* (dorsal aorta), and *flt4* (posterior cardinal vein). n=10 for all probes. Scale bars - 100 µm.

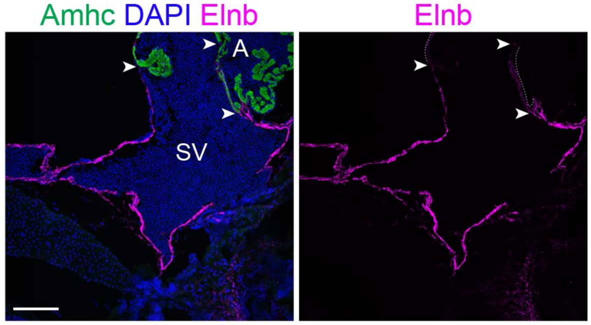

Supplementary Fig. 21. Overlap of atrial CMs and smooth muscle at the venous pole of the zebrafish heart. Confocal images of a mid-sagittal section from the venous pole of a WT heart showing that atrial CMs are surrounded by Elnb, suggesting the atrial CMs at the venous pole are encased by SMCs and that the CMs extended into the SV. Arrows and dashed lines indicate overlap of atrial CMs with Elnb at the atrial-SV junction. n=5 fish examined. Scale bar - 100 μm.

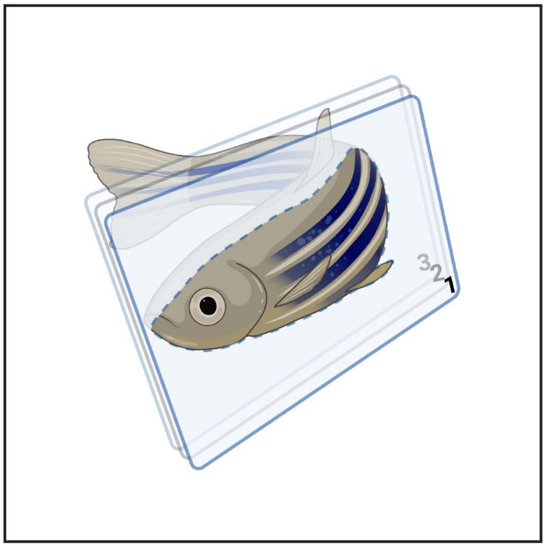

Supplementary Fig. 22. Schematic showing the location of sagittal sections in the midline of the fish used for analysis. Schematic created with BioRender.com.

Supplementary Table 1. Primers used.

	Name	Sequence
**Genotyping**	*amhc-p-F1*	CCCTTATTTGGCTCTGCGTTTG
	cre-g-R1	GCGAACCTCATCACTCGTTG
	nr2f1a-EcoRI-mut-F	GTTATATTGCAAAATCAGTTTAATC
	nr2f1a-g-R5	CAATGCTTGGAGTGTGAGGG
**qPCR**	*hoxb2a-F1*	AATCCTCGAAGAAGTGCCCC
	hoxb2a-R1	CCTGGGCCGACAAAGGTATT
	hoxb3a-F1	GCTCTGGGGGAACATCCAAA
	hoxb3a-R1	GCTCTCTTGGAAGCTGCAGA

Supplementary Table 2. Antibodies used

	Name	Host	Supplier	Product	Dilution
**Primary Antibodies**	anti-Sarcomeric myosin (Mhc)	Mouse (IgG2b)	DSHB	MF20	1:10
	anti-Myosin heavy chain, slow developmental (Amhc)	Mouse (IgG1)	DSHB	S46	1:10
	anti-GFP	Chicken	Invitrogen	A10262	1:250
	anti-Elnb	Rabbit	custom (YenZym.com)	Song et al. (2019)	1:250
	anti-Myosin light chain kinase	Mouse (IgG2b)	Sigma	M7905	1:250
	anti-RCFP polyclonal pan antibody	Rabbit	Clontech	632475	1:1000
	anti-Vmhc	Rabbit	custom (YenZym.com)	Song et al.(2019)	1:250
	anti-Digoxigenin-AP	Sheep	Roche	11093274910	1:5000
	anti-Nr2f1a	Rabbit	custom (YenZym.com)	Duong et al. (2018)	1:100
	anti-alpha-tubulin	Mouse (IgG1)	Sigma	T6199	1:1000
	Living Colors anti-DsRed antibody	Rabbit	Clontech	632496	1:1000

**Secondary Antibodies**	anti-Rabbit IgG(H+L) Alexa Fluor® 647	Goat	Southern Biotech	4050-31	1:200
	ant-Rabbit IgG(H+L) Alexa Fluor® 488	Goat	Southern Biotech	4050-30	1:200
	ant-Chicken IgY(H+L) Alexa Fluor® 488	Goat	Invitrogen	A11008	1:500
	anti-Mouse IgG2b TRITC	Goat	Southern Biotech	1090-03	1:200
	anti-Mouse IgG1 DyLight™ 405	Goat	BioLegend	409109	1:500
	anti-Mouse IgG1 TRITC	Goat	Southern Biotech	1070-03	1:200
	anti-Mouse IgG1 FITC	Goat	Southern Biotech	1070-02	1:200
	anti-Mouse IgG2b Alexa Fluor® 647	Goat	Southern Biotech	1091-31	1:200
	anti-rabbit secondary LI-COR IRDye 680LT	Donkey	LI-COR	926-68023	1:5000
	anti-mouse secondary LI-COR IRDye 800CW	Donkey	LI-COR	925-32212	1:5000
