# Supplementary material for: Sinus venosus adaptation models prolonged cardiovascular disease and reveals insights into evolutionary transitions of the vertebrate heart
Source: Nat Commun. 2023 Sep 7;14:5509. doi: 10.1038/s41467-023-41184-y (PMC10485058; doi:10.1038/s41467-023-41184-y)
Supplement: Supplementary file 3 — Description of Additional Supplementary Files [file 41467_2023_41184_MOESM3_ESM.pdf]

Description of Additional Supplementary Files

File Name: Supplementary Data 1
Description: **Differential Gene Expression Analysis (FD, P-Value, and FDR) of bulk RNA seq from the zebrafish BA, SV, V, and A.** P-value by Wald test using DESeq2, two-sided, adjustment made by FDR in a separate column.

File Name: Supplementary Data 2
Description: **Z-scores for hierarchical clustering analysis of bulk RNA seq fof the WT and nr2f1a mutant BA, SV, V, and A.**

File Name: Supplementary Data 3
Description: **Z-scores for hierarchical clustering of bulkd RNA seq from WT zebrafish BA, SV, V, and A.**

File Name: Supplementary Data 4
Description: **List of genes with similar expression in the WT BA and SV of adult zebrafish.**

File Name: Supplementary Data 5
Description: **Joint ICGS2 Cell Clusters.**

File Name: Supplementary Data 6
Description: **Marker genes for Clusters 1-12. MarkerFinder algorithm was used to determine the marker genes for each cluster.** MarkerFinder implements a two-sided Pearson correlation (rho) test between gene expression and an idealized lineage-specific gene expression profile (no expression in any samples except in the lineage of interest).

File Name: Supplementary Data 7
Description: **Gene-set enrichment comparing WT clusters top-200 genes to prior EC populations.** Gene-set enrichment analysis is performed using GO-Elite. A Z score and Fisher's Exact two-sided Test p-value are calculated to assess over-representation of Ontology terms, gene-sets, and pathways. Adjusted p-values (FDR p-values) for these various tests are calculated using the Benjamini-Hochberg correction method.

File Name: Supplementary Data 8
Description: **Organized differential gene expression for all clusters. Gene-set enrichment analysis is performed using GO-Elite.** A Z score and Fisher's Exact two-sided Test p-value are calculated to assess over-representation of Ontology terms, gene-sets, and pathways. Adjusted p-values (FDR p-values) for these various tests are calculated using the Benjamini-Hochberg correction method.

File Name: Supplementary Data 9
Description: **Differentially expressed genes (fold>1.2 and eBayes p<0.05, FDR) from nr2f1a mutant vs. WT SV.** A moderated two-sided t-test (unpaired, assuming equal variance), based on the limma empirical Bayes model, is implemented for the differential gene expression

analysis. Adjusted p-values (FDR p-values) for these various tests are calculated using the Benjamini-Hochberg correction method.

File Name: Supplementary Data 10
Description: **Gene-set enrichment comparing SV up- and down-regulated transcripts in each cluster to Gene Ontology terms.** Gene-set enrichment analysis is performed using GO-Elite. A Z score and Fisher's Exact two-sided Test p-value are calculated to assess over-representation of Ontology terms, gene-sets, and pathways. Adjusted p-values (FDR p-values) for these various tests are calculated using the Benjamini-Hochberg correction method.

File Name: Supplementary Data 11
Description: **GO-Elite gene-set enrichment of cellHarmony identified pattern specific genes for all significant DEGs (see Supplementary Tables 9 and 10).** A Z score and Fisher's Exact two-sided Test p-value are calculated to assess over-representation of Ontology terms, gene-sets, and pathways. Adjusted p-values (FDR p-values) for these various tests are calculated using the Benjamini-Hochberg correction method.

File Name: Supplementary Data 12
Description: **Ranked marker genes for all WT cells in BA and SV combined clusters.** MarkerFinder algorithm was used to determine the marker genes for each cluster. MarkerFinder implements a two-sided Pearson correlation (rho) test between gene expression and an idealized lineage-specific gene expression profile (no expression in any samples except in the lineage of interest).

File Name: Supplementary Data 13
Description: **Gene-set enrichment of WT clusters top-200 marker genes.** Gene-set enrichment analysis is performed using GO-Elite. A Z score and Fisher's Exact two-sided Test p-value are calculated to assess over-representation of Ontology terms, gene-sets, and pathways. Adjusted p-values (FDR p-values) for these various tests are calculated using the Benjamini-Hochberg correction method.

File Name: Supplementary Data 14
Description: **Gene-set enrichment comparing SV up- and down-regulated transcripts in each cluster to WT BA and SV top-200 marker genes.** Gene-set enrichment analysis is performed using GO-Elite. A Z score and Fisher's Exact two-sided Test p-value are calculated to assess over-representation of Ontology terms, gene-sets, and pathways. Adjusted p-values (FDR p-values) for these various tests are calculated using the Benjamini-Hochberg correction method.

File Name: Supplementary Data 15
Description: **Differentially expressed genes (fold>1.2 and eBayes p<0.05, FDR) in nr2f1a mutant vs. WT BA.** A moderated two-sided t-test (unpaired, assuming equal variance), based on the limma empirical Bayes model, is implemented for the differential gene expression analysis. Adjusted p-values (FDR p-values) for these various tests are calculated using the Benjamini-Hochberg correction method.

File Name: Supplementary Data 16
Description: **Gene-set enrichment comparing BA up- and down-regulated transcripts in each cluster to Gene Ontology terms.** Gene-set enrichment analysis is performed using GO-Elite. A Z score and Fisher's Exact two-sided Test p-value are calculated to assess over-representation of Ontology terms, gene-sets, and pathways. Adjusted p-values (FDR p-values) for these various tests are calculated using the Benjamini-Hochberg correction method.

File Name: Supplementary Data 17
Description: **Gene-set enrichment comparing BA up- and down-regulated transcripts in each cluster to WT top-200 marker genes.** Gene-set enrichment analysis is performed using GO-Elite. A Z score and Fisher's Exact two-sided Test p-value are calculated to assess over-representation of Ontology terms, gene-sets, and pathways. Adjusted p-values (FDR p-values) for these various tests are calculated using the Benjamini-Hochberg correction method.

File Name: Supplementary Data 18
Description: **DEG comparing c2 (wt) vs. c1 (mut) up- and down-regulation (eBayes t-test $p<0.05$, FDR corrected and fold>1.2).** A moderated two-sided t-test (unpaired, assuming equal variance), based on the limma empirical Bayes model, is implemented for the differential gene expression analysis. Adjusted p-values (FDR p-values) for these various tests are calculated using the Benjamini-Hochberg correction method.

File Name: Supplementary Data 19
Description: **Gene-set enrichment comparing c2 (wt) vs. c1 (mut) up- and down-regulated transcripts against GO, KEGG and WikiPathways.** Gene-set enrichment analysis is performed using GO-Elite. A Z score and Fisher's Exact two-sided Test p-value are calculated to assess over-representation of Ontology terms, gene-sets, and pathways. Adjusted p-values (FDR p-values) for these various tests are calculated using the Benjamini-Hochberg correction method.

File Name: Supplementary Data 20
Description: **Differential Gene Expression Analysis (FD, P-value, and FDR) of Ciona cardiac, pharyngeal blood sinus, and stomach blood sinus.** P-value by Wald test using DESeq2, two-sided, adjustment made by FDR in a separate column.

File Name: Supplementary Data 21
Description: **Z-scores of hierarchical clustering for Ciona C, PS, and SS RNA_seq samples.**

File Name: Supplementary Movie 1
Description: **(GIF) Echocardiography of an adult WT zebrafish heart.** Two-color Doppler video pseudo-coloring blood flow to allow for visualizing blood flow direction in adult WT zebrafish hearts. Orange indicates anterograde blood flow into the ventricle. Blue indicates retrograde blood flow away from the ventricle.

File Name: Supplementary Movie 2
Description: **(GIF) Echocardiography of a *nr2f1a* mutant zebrafish heart.** Two-color Doppler videos pseudo-coloring blood flow to allow for ease of visualizing flow direction in adult *nr2f1a*^{aco} mutant zebrafish. Orange indicates anterograde blood flow into the ventricle.

Blue indicates retrograde blood flow away from the ventricle, which shows flow into the SV of *nr2f1a*^{aco} mutants.

File Name: Supplementary Movie 3
Description: **(AVI) Juvenile zebrafish in the swim tunnel.** Juvenile *nr2f1a*^{aco} mutant swimming against a constant current to simulate exercise for 10 seconds.
