# Supplementary figures and images for: Sinus venosus adaptation models prolonged cardiovascular disease and reveals insights into evolutionary transitions of the vertebrate heart
Source: Nat Commun. 2023 Sep 7;14:5509. doi: 10.1038/s41467-023-41184-y (PMC10485058; doi:10.1038/s41467-023-41184-y)

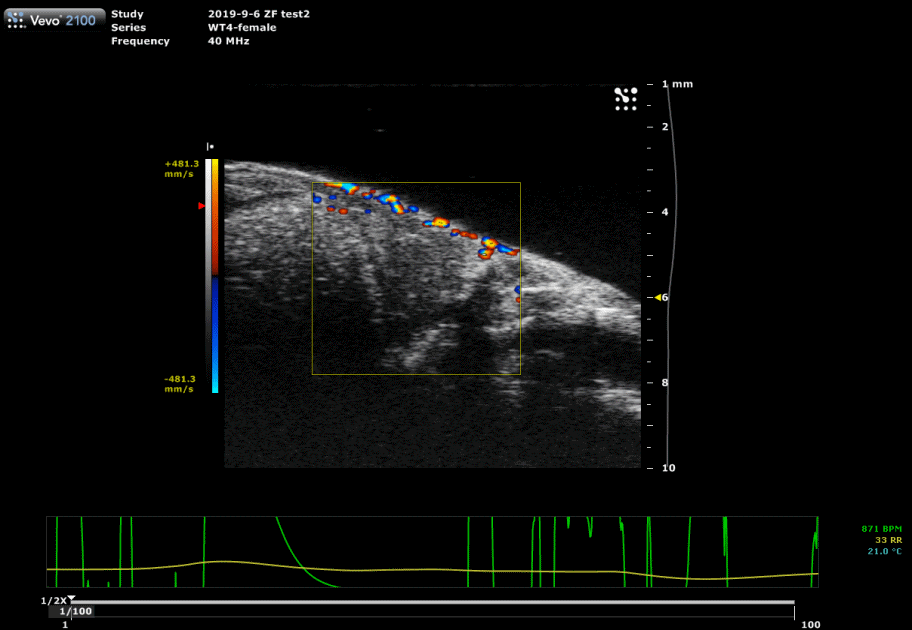

Supplement: Supplementary file 5 — Supplementary Movie 1 [file 41467_2023_41184_MOESM5_ESM.gif]

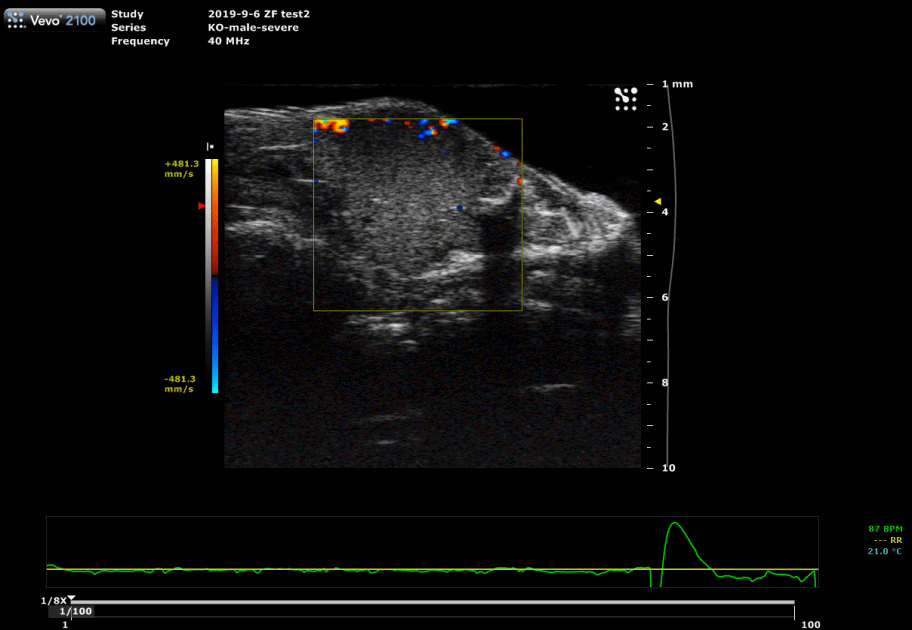

Supplement: Supplementary file 6 — Supplementary Movie 2 [file 41467_2023_41184_MOESM6_ESM.gif]
