# Supplementary material for: Sinus venosus adaptation models prolonged cardiovascular disease and reveals insights into evolutionary transitions of the vertebrate heart
Source: Nat Commun. 2023 Sep 7;14:5509. doi: 10.1038/s41467-023-41184-y (PMC10485058; doi:10.1038/s41467-023-41184-y)
Supplement: Supplementary file 8 — Reporting Summary [file 41467_2023_41184_MOESM8_ESM.pdf]

nature portfolio

Corresponding author(s): Joshua Waxman
Last updated by author(s): 8/15/2023

Reporting Summary

Nature Portfolio wishes to improve the reproducibility of the work that we publish. This form provides structure for consistency and transparency in reporting. For further information on Nature Portfolio policies, see our Editorial Policies and the Editorial Policy Checklist.

Statistics

For all statistical analyses, confirm that the following items are present in the figure legend, table legend, main text, or Methods section.

n/a	Confirmed	
☐	☒	The exact sample size (*n*) for each experimental group/condition, given as a discrete number and unit of measurement
☐	☒	A statement on whether measurements were taken from distinct samples or whether the same sample was measured repeatedly
☐	☒	The statistical test(s) used AND whether they are one- or two-sided<br>*Only common tests should be described solely by name; describe more complex techniques in the Methods section.*
☒	☐	A description of all covariates tested
☐	☒	A description of any assumptions or corrections, such as tests of normality and adjustment for multiple comparisons
☐	☒	A full description of the statistical parameters including central tendency (e.g. means) or other basic estimates (e.g. regression coefficient) AND variation (e.g. standard deviation) or associated estimates of uncertainty (e.g. confidence intervals)
☐	☒	For null hypothesis testing, the test statistic (e.g. *F*, *t*, *r*) with confidence intervals, effect sizes, degrees of freedom and *P* value noted<br>*Give P values as exact values whenever suitable.*
☒	☐	For Bayesian analysis, information on the choice of priors and Markov chain Monte Carlo settings
☒	☐	For hierarchical and complex designs, identification of the appropriate level for tests and full reporting of outcomes
☐	☒	Estimates of effect sizes (e.g. Cohen's *d*, Pearson's *r*), indicating how they were calculated

Our web collection on statistics for biologists contains articles on many of the points above.

Software and code

Policy information about availability of computer code

Data collection

Software used for acquisition of images: Zeiss Axiovision (v4.8), Zeiss Zen (v3.1) and Nikon Elements (v5.21.03). ImageJ(v1.53a) was used for measurements of histological sections.

Data analysis

Prism 9 was used for statistical analysis.

Bulk RNA-seq

Zebrafish bulk RNA-seq reads were aligned to the UCSC zebrafish genome, danRer11, using STAR aligner(v2.7.4) after trimming the Illumina adapter using cutadapt(v2.1). These libraries were prepped across multiple batches with different library types in terms of RNA strand and sequencing mode (single-end or paired-end). Therefore, we treated them as single-end libraries by choosing read1 or read2 to match the strand as much as possible, and batch correction was applied as described below. Only uniquely aligned reads were retained for downstream analysis. Gene expression levels were quantified as raw read counts using FeatureCounts in the subread(v1.6.2) package with an option, "-O --fracOverlap 0.8", and with a proper strand option (-s). Given the mixed sequencing batches and library types, we incorporated RUVseq(v1.28.0) batch correction (RUVs, k=1) in DESeq2(v1.34.0) differential analysis. Differentially expressed genes were identified by FDR < 0.05. Hierarchical clustering was performed using the Pearson correlation coefficient as a similarity measure under Ward's criterion, and a heatmap was visualized in z-score.

Ciona bulk RNA-seq data was analyzed in the same way as indicated above for zebrafish RNA-seq analysis except using the UCSC Ciona genome ci3. Differential genes were identified by P-value < 0.05 for hierarchical clustering.

Single cell RNA-seq

Single cells were submitted to the CCHMC Gene Expression Core for 10x Genomics analysis utilizing Chromium instrumentation. The scRNA-Seq FASTQ files (10x Genomics 3' version 3) were aligned to the Zebrafish Ensembl version 91 reference genome (GRCz11) and transcriptome, using Cell Ranger (v3.1.0). Cell Ranger filtered feature sparse matrix counts files from all samples (HDF5 format) were supplied to AltAnalyze148 (v2.1.4) for unsupervised analysis using the EnsMart 91 zebrafish database and ICGS2 algorithm with default parameters. Cell-type predictions were initially obtained from ICGS2 based on marker enrichment and then further refined based on manual curation with literature-associated markers. The obtained ICGS2 clusters were filtered to include muscle, endothelial and epithelial clusters for downstream UMAP visualization.

To confirm assignment of mutant cells to WT clusters, we performed supervised classification of all mutant capture cells to WT cells and clusters using the software cellHarmony. Supervised embedding of these mutant cells into a WT restricted UMAP was performed using scikit-learn to train and transform the ICGS2 marker genes using the top 50 PCA components (UMAP-transform). All differential expression analyses were performed using the software cellHarmony in AltAnalyze (fold>1.2 and empirical Bayes t-test p<0.05, FDR corrected), with secondary gene-set enrichment analyses and visualization performed in GO-Elite.

For pseudotime analysis, we applied both Monocle 2 and SlingShot. Log-transformed gene-by-cell expression file and cell type labels file from all samples generated from ICGS2 were filtered for smooth muscle cells (Clusters 1-8) and were provided as the input to Monocle 2(v2.13.0). The log-transformed file was exponentiated and modeled with negative binomial distribution using Monocle 2 ('expressionFamily=negbinomial.size'). Monocle 2 was allowed to select its own genes for pseudotime estimation based on differential gene analysis across the filtered smooth muscle ICGS2 groups ('fullModelFormulaStr = ~Groups'). The reverse graph embedding (RGE) method ('method' in reduceDimension) was set to "DDRTree" as recommended by the authors of Monocle 2. For SlingShot(v1.8.0), we embedded the gene expression data for the selected populations using the software SPRING, using programmatic defaults for both tools.

For manuscripts utilizing custom algorithms or software that are central to the research but not yet described in published literature, software must be made available to editors and reviewers. We strongly encourage code deposition in a community repository (e.g. GitHub). See the Nature Portfolio guidelines for submitting code & software for further information.

Data

Policy information about availability of data

All manuscripts must include a data availability statement. This statement should provide the following information, where applicable:

- Accession codes, unique identifiers, or web links for publicly available datasets
- A description of any restrictions on data availability
- For clinical datasets or third party data, please ensure that the statement adheres to our policy

RNA-seq data has been deposited in GEO. GEO accession numbers for RNA-seq data sets are: GSE195548, GSE195549, and GSE229821. All data are presented within the paper and association Supplemental Information and Source Data. There is no restriction on data availability.

Research involving human participants, their data, or biological material

Policy information about studies with human participants or human data. See also policy information about sex, gender (identity/presentation), and sexual orientation and race, ethnicity and racism.

Reporting on sex and gender	n/a
Reporting on race, ethnicity, or other socially relevant groupings	n/a
Population characteristics	n/a
Recruitment	n/a
Ethics oversight	n/a

Note that full information on the approval of the study protocol must also be provided in the manuscript.

Field-specific reporting

Please select the one below that is the best fit for your research. If you are not sure, read the appropriate sections before making your selection.

☒ Life sciences ☐ Behavioural & social sciences ☐ Ecological, evolutionary & environmental sciences

For a reference copy of the document with all sections, see nature.com/documents/nr-reporting-summary-flat.pdf

Life sciences study design

All studies must disclose on these points even when the disclosure is negative.

Sample size	Sample sizes for experiments were not determined prior to performing experiments. Samples sizes used in experiments are consistent with

Sample size	standards in the field due to practicality issues dictated by the time and number of animals needed for the experiments, type of experiments and age of the fish, and the experimental cost.
Data exclusions	No data were excluded from analysis of experiments reported in the manuscript.
Replication	All experiments were performed at least 2 times, except for the single-cell RNA-seq experiments. Replicates produced data that support the same conclusions. For bulk RNA-seq, experimental replicates from 20 pooled samples were used for analysis. A selection of differentially expressed genes from the hierarchical clustering were confirmed with immunohistochemistry. For Single-cell RNA-seq experiments, at least 24 tissue samples were pooled from each condition and tissue from an equal number of males and females to ensure the samples were representative of the tissue conditions. Similar changes in gene expression were found in the bulk and single-cell RNA-seq experiments supporting the reproducibility of the conditions.
Randomization	Experiments did not involved randomized samples.
Blinding	Blinding was not possible for experiments because they employed comparisons of wild-type and homozygous mutant fish. It was not possible to hide the identities of these samples from the experimenter or a blind analyzer due to overt morphological characteristics of the mutant fish, such as pericardial blood pooling and edema, and tissue samples, such as a lack of an atrium.

Reporting for specific materials, systems and methods

We require information from authors about some types of materials, experimental systems and methods used in many studies. Here, indicate whether each material, system or method listed is relevant to your study. If you are not sure if a list item applies to your research, read the appropriate section before selecting a response.

Materials & experimental systems

n/a	Involved in the study
☐	☒ Antibodies
☒	☐ Eukaryotic cell lines
☒	☐ Palaeontology and archaeology
☐	☒ Animals and other organisms
☒	☐ Clinical data
☒	☐ Dual use research of concern
☒	☐ Plants

Methods

n/a	Involved in the study
☒	☐ ChIP-seq
☒	☐ Flow cytometry
☒	☐ MRI-based neuroimaging

Antibodies

Antibodies used	anti-Amhc (Developmental Studies Hybridoma Bank; S46), anti-Mhc (Developmental Studies Hybridoma Bank, MF20), anti-Vmhc (custom generated; YenZym), anti-Elnb (custom generated; YenZym), anti-Mylk (Sigma; M7905), anti-RCFP (Clontech; 632475), anti-DsRed (Clontech; 632496), anti-Digoxigenin-AP (Roche; 11093274910), anti-Nr2f1a (custom generated; YenZym), anti-aTubulin (Sigma; T6199), anti-GFP (Invitrogen; A10262), anti-Rabbit IgG(H+L) Alexa Fluor® 647 (Southern Biotech; 4050-31), ant-Rabbit IgG(H+L) Alexa Fluor® 488 (Southern Biotech; 4050-30), ant-Chicken IgY(H+L) Alexa Fluor® 488 (Invitrogen; A11008), anti-Mouse IgG2b TRITC (Southern Biotech; 1090-03), anti-Mouse IgG1 DyLight™ 405 (BioLegend; 409109), anti-Mouse IgG1 TRITC (Southern Biotech; 1070-03), anti-Mouse IgG1 FITC (Southern Biotech; 1070-02), anti-Mouse IgG2b Alexa Fluor® 647 (Southern Biotech; 1091-31), anti-rabbit secondary LI-COR IRDye 680LT (LI-COR; 926-68023), anti-mouse secondary LI-COR IRDye 800CW (LI-COR; 925-32212)
Validation	Amhc antibody was validated in Stockdale, FE, JCB, 1985; Mhc antibody was validated in Fischman, DA, JCB, 1982; Vmhc antibodywas validated in Song et al, 2019 based on established expression Vmhc in the heart; Elnb antibody was validated in Song et al, 2019 based on established expression in the bulbus arteriosus in the outflow tract; , Mylk antibody has been used in 64 papers according to supplier site; RCFP antibody was validated by Western Blot on supplier site; DsRed antibody was validated by Western Blot on supplier site, Digoxigenin-AP antibody has been used in 707 publication according to supplier website, Nr2f1a antibody was validated via Western blot and immunohistochemistry in Duong T, Dev Bio, 2018; aTubulin antibody has been used in 2077 papers according to supplier; GFP antibody has been used in 319 papers according to supplier site.

Animals and other research organisms

Policy information about studies involving animals; ARRIVE guidelines recommended for reporting animal research, and Sex and Gender in Research

Laboratory animals	Zebrafish (Danio rerio) of mixed AB/TL lineage were used. Transgenic lines used in this study were: Tg(amhc:Cre-ERT2)sd20, Tg(ubb:loxP-AmCyan-loxP-ZsYellow)fb5, Tg(sox10:GAL4,UAS:Cre)la2326, Tg(-3.5ubb:loxP-EGFP-loxP-mCherry)cz1701, Tg(acta2:eGFP)ca7, and Tg(kdrl:GFP)s843. The zebrafish mutant allele nr2f1aacoci1017 was used in this study. Zebrafish were used at ages from 48 hours post-fertilization through 1 year old (adults). Adult fish 2.0-2.5 cm were used for analysis in experiments.
Wild animals	Ciona robusta (formerly called Ciona intestinalis type A) were used in the study. Adult Ciona robusta estimated to be 5-7 months old with a size ranging from 5 to 10 cm were collected in the Gulf of Taranto, Italy, by hand picking at low depth. All collected Ciona were used in the study. The Ciona died after the surgery as it removed their heart, which is necessary for survival.

Reporting on sex	Sex was recorded when performing experiments, but not reported as differences were not found.
Field-collected samples	Wild caught Ciona robusta were transported to the Stazione Zoologica Anton Dohrn in Napoli (SZN) with an animal transport van equipped with refrigerated tanks and supplied with oxygen air pump. Once they arrived at the Stazione Zoologica the animals were maintained in the animal facility, an open tank system of 150-200 Liters, with parameters: ~20°C , pH ~7.8, salinity ~37.9 ‰ and with a photoperiod of 12L:12D. Animals were fed every day with a solution of shellfish diet 1800TM Instant Algae®.
Ethics oversight	All work with zebrafish carried out in accordance with guidelines established by Cincinnati Children's Hospital Medical Center (CCHMC) and protocol IACUC 2020-0091, approved by the CCHMC Institutional Animal Care and Use Committee (IACUC). Adult zebrafish were monitored daily by veterinary technicians at CCHMC.<br><br>Invertebrate organisms in Italy are not subject to institutional care committees as Ciona robusta, a marine invertebrate, are not included in the law of DIRECTIVE 2010/63/EU OF THE EUROPEAN PARLIAMENT AND OF THE COUNCIL of 22 September 2010, which details the protection of animals used for scientific purposes. Ciona robusta as invertebrate animals are considered to not have the ability to experience discernible pain, suffering, distress, and lasting harm.

Note that full information on the approval of the study protocol must also be provided in the manuscript.
